# The effects of vitamin D supplementation on muscle function among postmenopausal women: a systematic review and meta-analysis of randomized controlled trials

**DOI:** 10.17179/excli2019-1386

**Published:** 2019-08-06

**Authors:** Reza Tabrizi, Jamal Hallajzadeh, Naghmeh Mirhosseini, Kamran B Lankarani, Najmeh Maharlouei, Maryam Akbari, Zatollah Asemi

**Affiliations:** 1 Health Policy Research Center, Institute of Health, Shiraz University of Medical Sciences, Shiraz, Iran; 2 Clinical Neurology Research Center, Shiraz University of Medical Sciences Shiraz, Iran; 3 Department of Biochemistry and Nutrition, Research Center for Evidence-Based Health Management, Maraghe University of Medical Science, Maraghe, Iran; 4 School of Public Health, University of Saskatchewan, Saskatoon, Canada; 5 Research Center for Biochemistry and Nutrition in Metabolic Diseases, Kashan University of Medical Sciences, Kashan, Iran

**Keywords:** vitamin D supplementation, muscle function, postmenopausal women, meta-analysis

## Abstract

The loss of muscle mass and its strength is one of the most critical changes in aging which is associated with an increased risk of falls, osteoporotic fractures and mobility disability. Vitamin D, with its extra-skeletal benefits, might improve muscle function in elderly. The current systematic review and meta-analysis of randomized controlled trials (RCTs) was conducted to summarize available relevant data and determine the effect of vitamin D supplementation on muscle function among postmenopausal women. We reached databases including; Cochrane library, Embase, PubMed, and Web of Science database until the end of May 2018 to identify relevant published RCTs. Heterogeneity among included studies was assessed using Q-test and I^2^ statistics. Random-effect model was applied to pool data and weighted mean difference (WMD) was calculated representing summary effect size. Outcomes of interest included the effects of vitamin D supplementation on hand grip strength (HGS), back muscle strength (BMS), and Timed Up and Go (TUG). Twelve RCTs out of 1739 potential reports were included in our meta-analysis. The pooled findings showed that vitamin D supplementation had no significant effect on HGS (WMD -0.03 kilogram (Kg); 95 % CI, -0.26, 0.20; P=0.78), BMS (WMD 7.21 newton (N); 95 % CI, -5.98, 20.40; P=0.28), and TUG (WMD 0.01 second (S); 95 % CI, -0.17, 0.18; P=0.93) in postmenopausal women. Overall, the current meta-analysis showed that taking vitamin D supplementation by postmenopausal women did not affect markers of muscle function. Further studies are required to confirm the effect of vitamin D supplementation on markers of muscle function.

## Introduction

Muscle function is one of the main determinants of functional capacity among postmenopausal women. Lack of muscle mass and muscle strength occurs as important organic changes by aging and is remarkably associated with an elevated risk of falls and subsequent osteoporotic fractures, besides this severely influences the potential of movement (Orsatti et al., 2011[26]; Muir and Montero-Odasso, 2011[25]). Vitamin D deficiency is common among older populations worldwide, as more than 60 % of postmenopausal women are vitamin D deficient, regardless of other nutrient deficiencies (Rizzoli et al, 2006[30]; Holick, 2007[20]; Han et al., 2018[17]). In observational studies, optimal serum 25(OH)D levels have been significantly associated with improved musculoskeletal function and muscle strength (Bischoff-Ferrari et al., 2004[6]; Visser et al., 2003[36]). Further, postmenopausal women who were vitamin D deficient presented worse upper and lower limb muscle strength and physical performance compared to vitamin D sufficient control group (Iolascon et al., 2015[21]). Skeletal muscle cells have vitamin D receptor and adequate protein synthesis in these cells, which might be directed through vitamin D function (Holick, 2007[20]). 

Current evidence evaluating the effects of vitamin D supplementation on musculoskeletal health has demonstrated promising results. In a meta-analysis conducted by Rosendahl-Riise et al. (2017[31]), vitamin D supplementation alone or its co-administration with calcium supplements significantly improved muscle strength among community-dwelling older individuals. In another meta-analysis, consuming vitamin D by healthy people significantly increased upper and lower limb strength (Tomlinson et al., 2015[34]). Moreover, Rejnmark (2011[29]) reported the advantages of vitamin D supplementation on muscle strength and function in older adults. In another trial, taking vitamin D by postmenopausal women increased their muscle strength, controlled lean mass loss and protected them against the occurrence of sarcopenia (loss of skeletal muscle mass) (Cangussu et al., 2015[10]). One-year treatment with high-dose vitamin D did not show any significant effect on muscular strength, balance or quality of life in postmenopausal osteoporotic women (Grimnes et al., 2017[16]). 

Despite existing published RCTs evaluating the effect of vitamin D on muscle function, current evidence is inconclusive because of lack of trials conducted among younger populations and discrepant findings in older individuals. Discrepancies in current findings might be due to the differences in study design, study populations' characteristics, the dosage of vitamin D supplementation, the duration of intervention, co-supplementation with other nutrients and chronic comorbid conditions among elderly. This meta-analysis was conducted to summarize available data and to determine the effect of vitamin D supplementation on muscle function among postmenopausal women.

## Methods

### Search strategy and study selection 

Two independent authors systematically searched databases including PubMed, EMBASE, Scopus, Cochrane Library, and Web of Science databases to identify RCTs investigating the impact of vitamin D on muscle function outcomes. Included trials were restricted to human clinical trials published in English until May 2018. The following MeSH and text terms were used to determine related studies, using their title and abstract: participants ["post menopause" OR "postmenopausal" OR "PMP"], intervention ("ergocalciferol" OR "alphacalcidol" OR "cholecalciferol" OR "eldecalcitol" OR "alfacalcidol" OR "vitamin D" OR "vitamin D2" OR "vitamin D3" AND "intake" OR "supplementation"), and outcomes ["hand grip strength (HGS)" OR "back muscle strength (BMS)" OR "Timed Up and Go (TUG)"]. 

To increase the possibility of including more related studies, the reference list of selected studies and previously published relevant reviews were manually searched. 

### Inclusion and exclusion criteria

First, duplicate studies and those clearly were not relevant were removed from further investigation. Then, the full-texts of remaining studies were retrieved to assess whether these trials met the inclusion criteria for the current meta-analysis. The inclusion criteria were as followed: being a human RCTs (either parallel or cross-over method), study population were post-menopause women, being a placebo-controlled trial, mean difference and standard deviations (SDs), standard error of the mean (SEMs), or 95 % confidence intervals (CIs) were available for considered outcomes including HGS, BMS, and TUG at both baseline and end of the intervention for each group. Observational studies, case reports, animal, and *in vitro* studies, and RCTs did not meet the minimum requirement for the assessment of methodological quality were excluded from this meta-analysis.

### Data extraction and quality assessment

The quality of selected RCTs methodology was assessed, using Cochrane Collaboration risk of bias tool and the data were extracted, using standardized excel sheet, by two independent authors (JH and MA). The following items were used to assess the quality of selected clinical RCTs and the probable risk of bias: "randomization generation, allocation concealment, blinding of participants and outcome assessment, incomplete outcome data, and selective outcome reporting, and other sources of bias". The following data were extracted from each study: first author's name, publication year, participants' age, study design and location, sample size in treatment and placebo groups, dosage of intervention, type of vitamin D supplement and placebo, and the mean (SD) changes for muscle outcomes in each group. If muscle outcomes were measured at multiple durations of intervention or using different dosages of vitamin D supplementation in selected studies, those measurements were considered as separate trials in meta-analysis. Any discrepancy in data extraction was resolved with consensus or by discussing with a third author (ZA).

### Data synthesis and statistical analysis

Weighted mean differences (WMDs) were used to determine the impacts of vitamin D supplementation on muscle outcomes including HGS, BMS, and TUG for each clinical trial. The changed value approach was applied to estimate the pooled effect sizes for each outcome. Authors evaluated existing heterogeneity across included RCTs, using Cochrane's Q test and I-square (I^2^). Heterogeneity was defined as the I^2 ^more than 50 percent with P <0.05. Upon existing heterogeneity, random-effect model was applied, using DerSimonian and Laird method; otherwise a fixed-effect model with inverse variance method was used to calculate the pooled WMDs with 95 % confidence interval (CI). Further, sensitivity and subgroup analyses were conducted to calculate the effects of each study on pooled WMDs and to examine the source of heterogeneity, using the possible moderator variables, respectively. P value < 0.05 was considered as statistically significant level. Statistical analyses were performed using STATA software version 12.0 (Stata Corp., College Station, TX) and RevMan V.5.3 software (Cochrane Collaboration, Oxford, UK). 

## Results

### Description of the selected RCTs 

**Figure 1:**
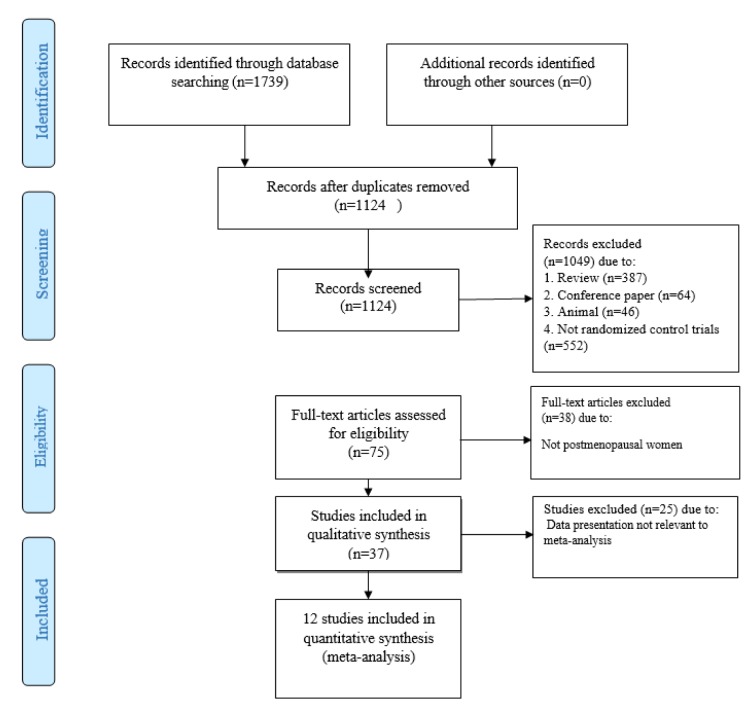
Flowchart of literature search for selection of trials

Initially, literature search identified 1739 potential studies. After screening based on keywords, removing duplicates and assessing the quality of identified papers, twelve articles (or 16 trials) were included in the meta-analysis. The detailed flowchart of step-by-step identification and selection of the RCTs following literature review has been illustrated in Figure 1(Fig. 1). The main characteristics of selected RCTs have been summarized in Table 1(Tab. 1) (References in Table 1: Anek et al., 2015[1]; Brunner et al., 2008[9]; Cangussu et al., 2015[10]; Cavalcante et al., 2015[11]; Gao et al., 2015[13]; Glendenning et al., 2012[14]; Hansen et al., 2015[18]; Hara et al., 2013[19]; Janssen et al., 2010[22]; Saito et al., 2016[32]; Wood et al., 2014[38]; Zhu et al., 2010[39]). 

Overall in 16 trials, 7765 subjects (3788 individuals in intervention and 3977 in control groups) were included in the meta-analysis. Eleven RCTs reported the effects of vitamin D on HGS (kilogram (Kg)), three on BMS (newton (N)), and six on TUG (secon (S)). This meta-analysis included RCTs published from 2000 till 2018. Duration of intervention and the dosages of vitamin D supplement varied from 12 to 96 weeks and 10 to 50,000 IU/day, respectively.

### Risk of bias and publication bias

**Figure 2:**
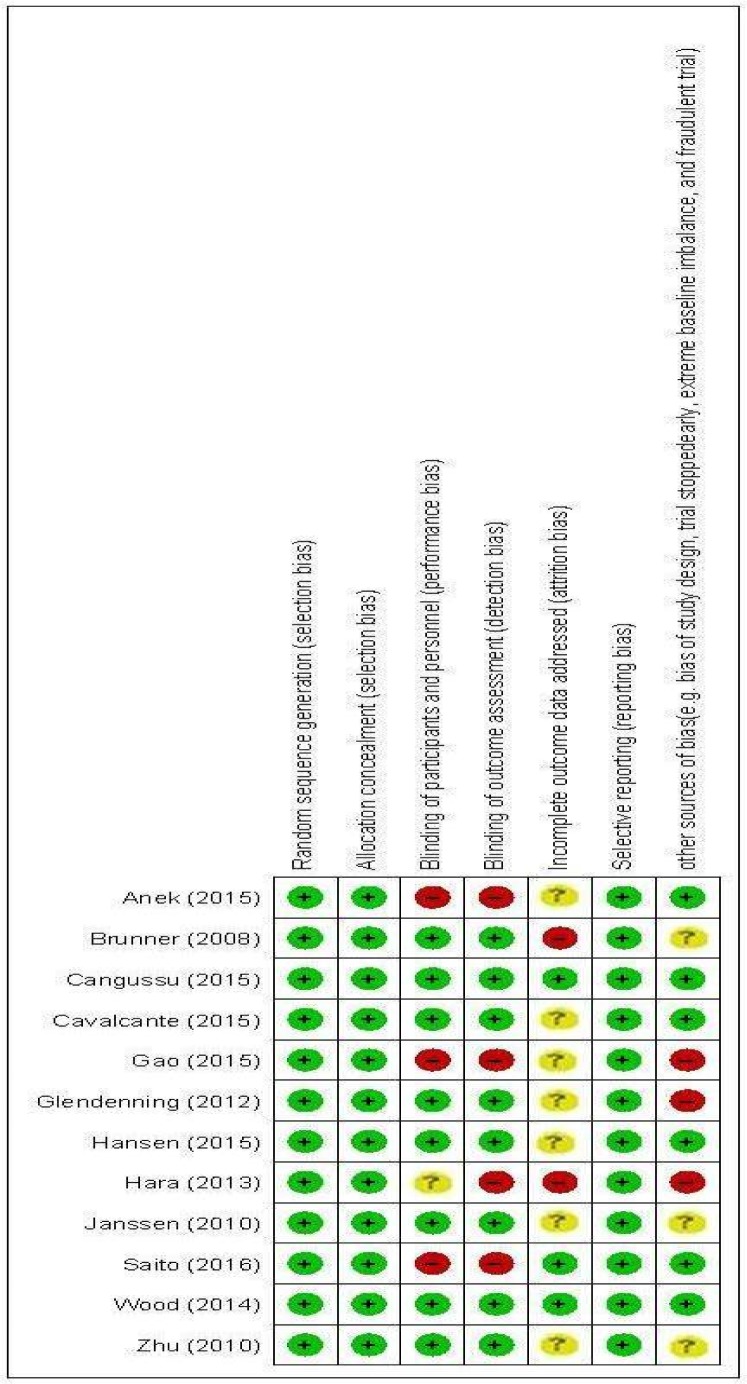
The methodological quality of included trials (risk of bias)

Egger and Begg's statistics were used to assess the possibility of publication bias across included RCTs. Our findings indicated no significant evidence of publication bias for studies included in the meta-analyses evaluating the impact of vitamin D supplementation on HGS (P Egger's test=0.48, P Begg's test= 0.58) and BMS (P_Eg_=0.71, P_Be_= 0.60). We found publication bias for TUG (P_Eg_=0.02, P_Be_= 0.18), therefore non-parametric method (Duval and Tweedie) was applied to examine the censored study findings. However, the results showed that overall pooled WMD for TUG did not significantly vary between pre (WMD 0.01 S; 95 % CI, -0.17, 0.18) and post (WMD 0.01 S; 95 % CI, -0.17, 0.18) including censored RCTs., the methodological quality of the included RCTs was judged, following Cochrane Handbook for Systematic Reviews of Interventions, by authors and the findings for different items have been illustrated in Figure 2(Fig. 2) (References in Figure 2: Anek et al., 2015[1]; Brunner et al., 2008[9]; Cangussu et al., 2015[10]; Cavalcante et al., 2015[11]; Gao et al., 2015[13]; Glendenning et al., 2012[14]; Hansen et al., 2015[18]; Hara et al., 2013[19]; Janssen et al., 2010[22]; Saito et al., 2016[32]; Wood et al., 2014[38]; Zhu et al., 2010[39]).

### Main outcomes, muscle function

**Figure 3:**
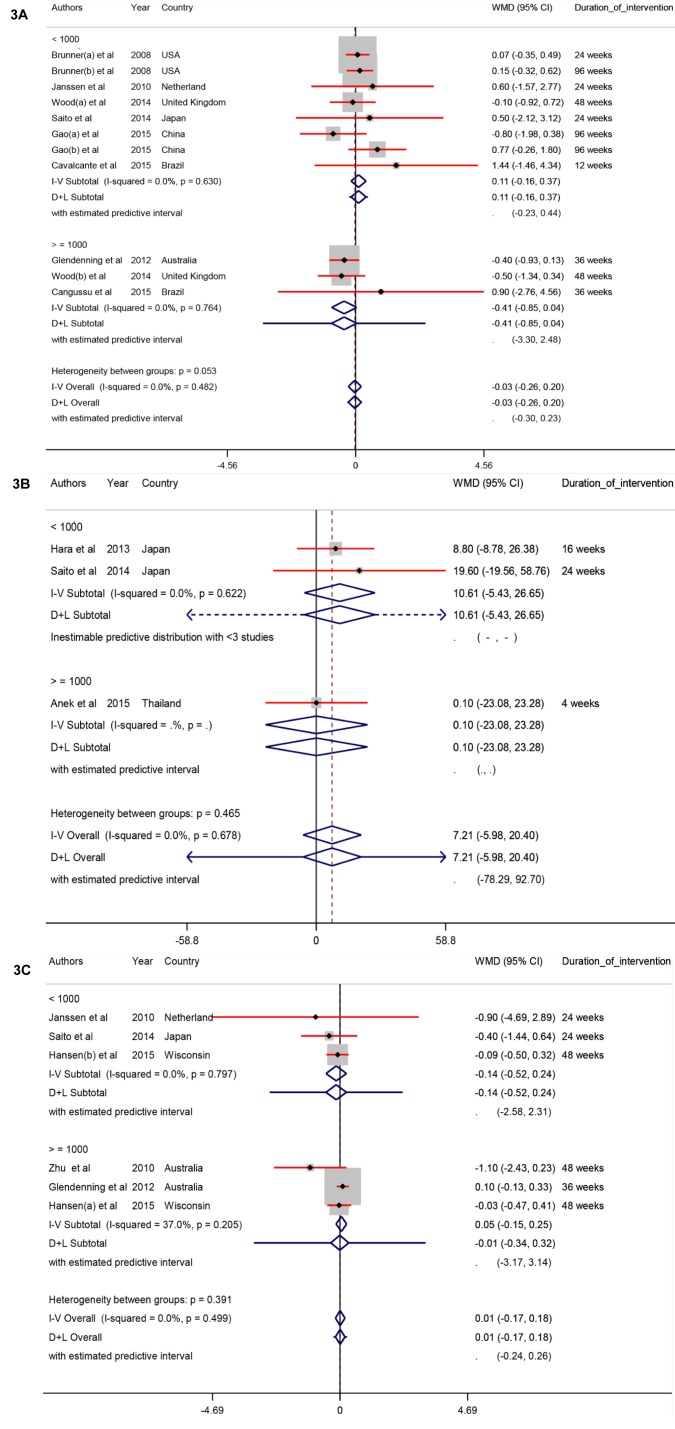
Figure 3A-C: Meta-analysis on muscle outcomes weighted mean differences estimates for (A) HGS, (B) for BMS, and (C) for TUG in vitamin D and placebo groups (CI=95 %)

The pooled results of included RCTs indicated that vitamin D supplementation did not affect any significant effect on HGS (WMD -0.03 Kg; 95 % CI, -0.26, 0.20; P=0.78), BMS (WMD 7.21 N; 95 % CI, -5.98, 20.40; P=0.28), and TUG (WMD 0.01 S; 95 % CI, -0.17, 0.18; P=0.93) in postmenopausal women (Table 2(Tab. 2) and Figure 3A-C(Fig. 3); References in Figure 3: Anek et al., 2015[1]; Brunner et al., 2008[9]; Cangussu et al., 2015[10]; Cavalcante et al., 2015[11]; Gao et al., 2015[13]; Glendenning et al., 2012[14]; Hansen et al., 2015[18]; Hara et al., 2013[19]; Janssen et al., 2010[22]; Saito et al., 2016[32]; Wood et al., 2014[38]; Zhu et al., 2010[39]).

### Subgroups and sensitivity analyses

The findings of subgroup analyses for the effect of vitamin D supplementation on muscle outcomes have been summarized in Table 3(Tab. 3).

Sensitivity analysis was applied to assess the impact of each study on the reliability of the association between vitamin D supplementation and muscle function. After excluding each study from the analyses, we observed no considerable difference between the pre- and post-sensitivity pooled WMD for HGS, BMS, and TUG. However, the lower and upper pooled WMD for HGS in the sensitivity analysis were -0.09 Kg (95 % CI: -0.35, 0.17) after omitting Brunner_(b)_ et al. (2008[9]) and 0.05 Kg (95 % CI: -0.20, 0.30) after omitting Glendenning et al. (2012[14]), respectively. For BMS, the lower WMD in the sensitivity analysis was 5.15 N (95 % CI: -14.78, 25.10) after excluding Hara et al. (2013[19]) and upper pooled WMD was 10.61 N (95 % CI: -5.42, 26.64) after excluding Anek et al. (2015[1]). The lower WMD for TUG was -0.13 S (95 % CI: -0.41, 0.14) after omitting Glendenning et al. (2012[14]) and upper pooled WMD was 0.02 S (95 % CI: -0.15, 0.20) after omitting Zhu et al. (2010[39]). 

## Discussion

To our best knowledge, this systematic review and meta-analysis was the first report determining the effect of vitamin D supplementation on muscle function among postmenopausal women. This meta-analysis showed that taking vitamin D supplementation by postmenopausal women did not affect markers of muscle function. 

Current evidence demonstrating the impact of vitamin D supplementation on lower extremity muscle strength and function are controversial. While some trials have confirmed a significant effect of vitamin D administration on improving lower extremity muscle strength and function in older population (Gloth et al., 1995[15]; Verhaar et al., 2000[35]), others did not (Kenny et al., 200[23]3; Latham et al, 2003[24]). Most of these studies included males and females who were not vitamin D deficient. In a study conducted by Zhu et al. (2010[39]), 1,000 IU/day vitamin D supplementation for 1 year significantly improved muscle strength in subjects who had low baseline muscle strength and whose serum 25(OH)D levels were below 24 ng/mL. TUG represents basic mobility skills, it is an effective method and evaluates functional mobility in older population (Podsiadlo and Richardson, 1991[28]). It is also a sensitive and specific measure for identifying the risk of fall among community-dwelling adults older than 65 years (Shumway-Cook et al., 2000[33]). Further, vitamin D ingestion was shown to improve neuromuscular coordination in older adults who had a history of fall with serum 25(OH)D levels less than 30 nmol/L (12 ng/mL) (Dhesi et al., 2004[12]). So, TUG improvement could be correlated with improved muscle strength and neuromuscular function. In a meta-analysis conducted by Beaudart et al. (2014[3]), vitamin D supplementation had a modest positive impact on muscle strength. In another meta-analysis, vitamin D supplementation to healthy individuals increased upper and lower limb strength (Tomlinson et al., 2015[34]). On the other hand, Rosendahl-Riise et al. (2017)[31] did not observe any improvement in muscle strength following vitamin D supplementation (with or without calcium supplements) in community-dwelling older population in their meta-analysis. Epidemiological studies have proposed serum 25(OH)D levels greater than 20 ng/mL as a desirable level for optimal lower extremity strength (Wicherts et al., 2007[37]; Bischoff-Ferrari et al., 2004[8]). The dosages of vitamin D used, type of vitamin D and the duration of intervention are some possible reasons which might explain the inconclusive results regarding the impacts of vitamin D on muscle function.

Low circulating levels of vitamin D is usually asymptomatic, however patient may present with loss of muscle mass and muscle weakness (Bergman et al., 2010[4]). Muscle cells have vitamin D receptors (VDR) (Bischoff-Ferrari et al., 2009[7]). Vitamin D metabolites can influence muscle cell metabolism through activating gene transcription, variation in the VDR allele, and rapid pathways not involved in DNA synthesis (Bischoff-Ferrari et al., 2009[7]; Barr et al., 2010[2]). Supplementation with vitamin D might enhance appendicular muscle strength and increase physical function, particularly in vitamin D deficient older population (Bischoff et al., 2003[5]; Pfeifer et al., 2009[27]).

The current study had a few limitations. We found a significant heterogeneity across included trials which might be explained through the large number of studies included and the variability observed between different protocols of supplementation. However, we considered this heterogeneity by applying a random effect model in meta-analyses. We were also unable to find any dose-response effect in this meta-analysis, which is probably due to the variability of the different supplementation protocols across included studies. 

## Conclusions

Overall, the current meta-analysis showed that taking vitamin D supplementation by postmenopausal women did not affect markers of muscle function. Further studies are required to confirm the effect of vitamin D supplementation on markers of muscle function. 

## Declarations

### Ethics approval and consent to participate

All procedures performed in studies involving human participants were in accordance with the ethical standards of the institutional and national research committee and with the 1964 Helsinki declaration and its later amendments.

### Consent for publication

Not applicable.

### Availability of data and material

The primary data for this study is available from the authors on direct request.

### Conflict of interest

Reza Tabrizi, Jamal Hallajzadeh, Naghmeh Mirhosseini, Kamran B Lankarani, Najmeh Maharlouei, Maryam Akbari and Zatollah Asemi declare that they have no conflict of interest.

### Funding

The research grant (97-01-106-17275) was provided by Research Deputy of Shiraz University of Medical Sciences (SUMS).

### Author contributions

ZA, JH, MA and RT contributed in conception, design, statistical analysis and drafting of the manuscript. NM, K-BL and NM contributed in conception, data collection and manuscript drafting. MM, NM and MC contributed in revised version. The final version was confirmed by all authors for submission. 

### Acknowledgements

The present study was supported by a grant from the Vice-chancellor for Research, SUMS, Shiraz, and Iran. 

## Figures and Tables

**Table 1:**
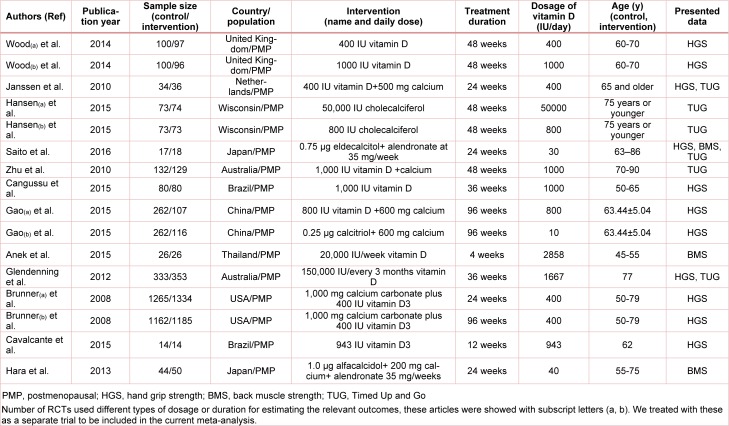
Characteristics of included studies

**Table 2:**
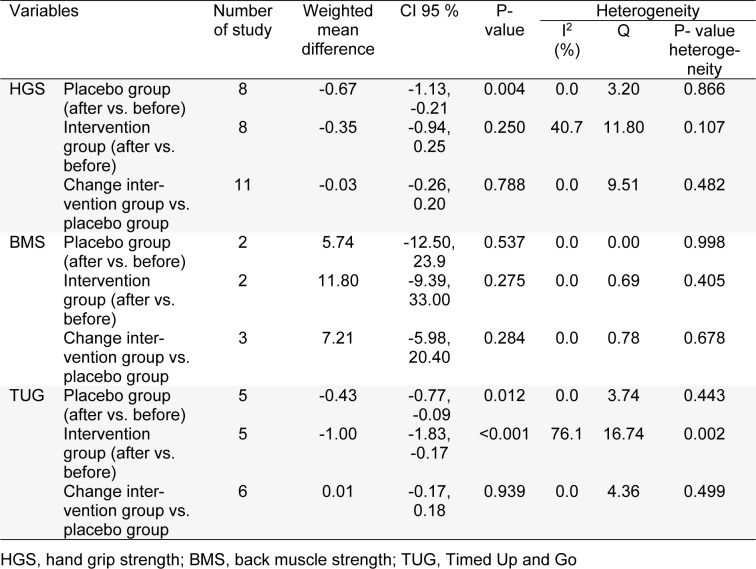
The effects of vitamin D supplementation on muscle strength in postmenopausal women

**Table 3:**
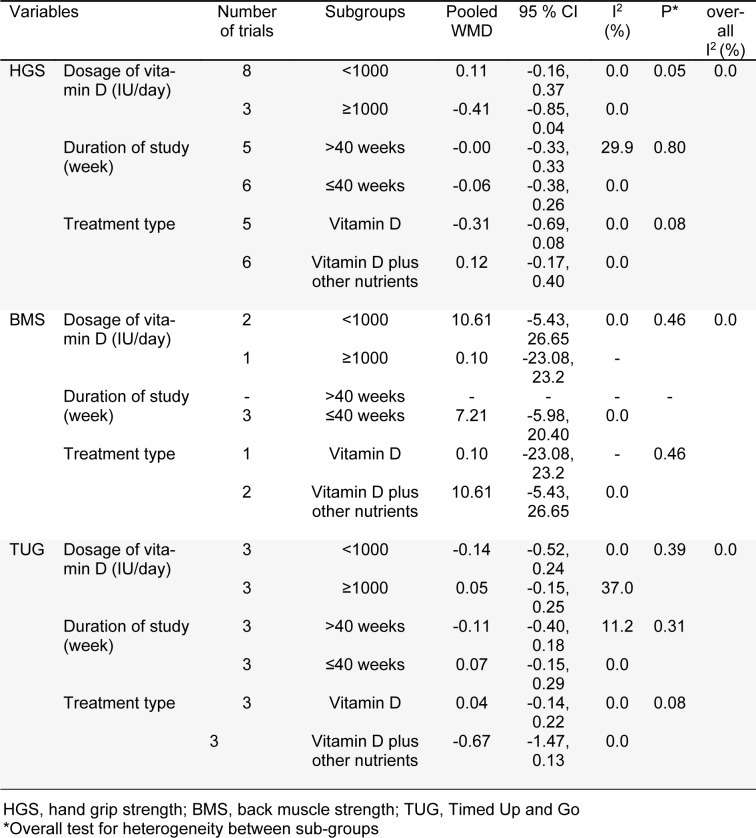
The association between vitamin D supplementation on muscle strength in postmenopausal women based on subgroup analysis
